# Asciminib vs bosutinib in CML patients pretreated with ≥2 tyrosine kinase inhibitors: Results from the Japanese subgroup analysis of ASCEMBL study

**DOI:** 10.1002/cam4.5212

**Published:** 2022-09-27

**Authors:** Junichiro Yuda, Noriko Doki, Hiroshi Matsuoka, Takafumi Yokota, Akihiro Tomita, Naoto Takahashi, Itaru Matsumura, Kohmei Kubo, Tatsunori Goto, Keita Kirito, Akio Maki, Makoto Aoki, Alex Allepuz, Yosuke Minami

**Affiliations:** ^1^ National Cancer Center Hospital East Chiba Japan; ^2^ Tokyo Metropolitan Cancer and Infectious Diseases Center Komagome Hospital Tokyo Japan; ^3^ Kobe University Hospital Kobe Japan; ^4^ Osaka University Hospital Osaka Japan; ^5^ Fujita Health University School of Medicine Toyoake Japan; ^6^ Akita University Hospital Akita Japan; ^7^ Kindai University Hospital Osaka Japan; ^8^ Aomori Prefectural Central Hospital Aomori Japan; ^9^ Japanese Red Cross Aichi Medical Center Nagoya Daiichi Hospital Nagoya Japan; ^10^ University of Yamanashi Hospital Yamanashi Japan; ^11^ Novartis Pharma KK Tokyo Japan; ^12^ Novartis Pharma AG Basel Switzerland

**Keywords:** ASCEMBL, asciminib, BCR‐ABL1 inhibitor, chronic myeloid leukemia, major molecular response, STAMP, tyrosine kinase inhibitors

## Abstract

Asciminib, a first‐in‐class, allosteric inhibitor of BCR‐ABL1 that acts by STAMP (Specifically Targeting the ABL Myristoyl Pocket), is a novel therapeutic option for patients with chronic myeloid leukemia (CML). In the global, phase 3, open‐label ASCEMBL study in patients with CML in chronic phase (CML‐CP) pretreated with ≥2 tyrosine kinase inhibitors (TKIs) (NCT03106779), asciminib (40 mg twice‐daily) demonstrated significant superiority over the ATP‐competitive TKI bosutinib (500 mg once daily) for the primary endpoint of major molecular response (MMR; *BCR::ABL1* transcript levels on the international scale [*BCR::ABL1* ^IS^] ≤0.1%) at week 24. Here, we report results from a descriptive subgroup analysis of Japanese patients enrolled in ASCEMBL study (data cut‐off: May 25, 2020). Overall, 16 Japanese patients were randomized (asciminib, *n* = 13; bosutinib, *n* = 3). At week 24, the MMR rate with asciminib was 30.8% (4/13; 95% confidence interval [CI], 9.09–61.43). *BCR::ABL1* ^IS^ ≤1% and complete cytogenic response (CCyR) at week 24 were 61.5% (8/13 patients) and 50.0% (4/8 patients), respectively. In the bosutinib group, no patient achieved MMR, CCyR, or *BCR::ABL1* ^IS^ ≤1%, but results were limited by the low number of patients. The safety profile of asciminib was comparable to that previously observed in the overall study population. Findings from this Japanese subgroup analysis of the ASCEMBL study support the use of asciminib for the treatment of Japanese patients with CML‐CP previously treated with ≥2 TKIs. ClinicalTrials.gov Identifier: NCT03106779.

## Graphical abstract

Asciminib, a first‐in‐class, allosteric inhibitor of BCR‐ABL1 that acts by STAMP (Specifically Targeting the ABL Myristoyl Pocket), is a novel therapeutic option for patients with chronic myeloid leukemia (CML). In the global, phase 3, open‐label ASCEMBL study in patients with CML in chronic phase (CML‐CP) pretreated with = 2 tyrosine kinase inhibitors (TKIs), asciminib (40 mg twice‐daily) demonstrated statistically significant superiority over the ATP‐competitive TKI bosutinib (500 mg once daily) for the primary endpoint of major molecular response (MMR; BCR‐ABL1 transcript levels on the international scale [BCR‐ABL1IS] = 0.1%) at week 24. Here, we present a descriptive subgroup analysis results of Japanese patients enrolled in the ASCEMBL study.

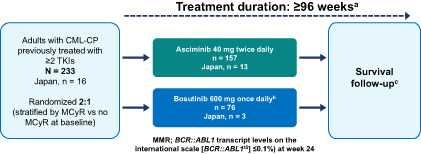

## 1 INTRODUCTION

Chronic myeloid leukemia (CML) is a myeloproliferative neoplasm defined by the presence of the Philadelphia chromosome (Ph), which is a result of reciprocal translocation of the Abelson (*ABL*) gene on chromosome 9 and the breakpoint cluster region (*BCR*) gene on chromosome 22. This fusion leads to the oncogenic *BCR::ABL1* gene, which encodes the BCR‐ABL1 oncoprotein.^1^, ^2^ Tyrosine kinase inhibitors (TKIs) are recommended treatment options for patients with CML,^3^, ^4^, ^5^, ^6^ with all currently approved TKIs targeting the adenosine triphosphate (ATP) binding site of ABL1.^3^, ^4^, ^5^, ^6^ Although many patients initially respond to first‐line TKIs,^7^ some patients eventually experience resistance or intolerance and fail on these therapies.^8^

TKIs currently available in Japan include the first‐generation TKI imatinib, the second‐generation TKIs nilotinib, dasatinib, and bosutinib, and the third‐generation TKI ponatinib.^6^, ^9^, ^10^, ^11^ Of these, imatinib, nilotinib, dasatinib, and recently bosutinib are approved in Japan for both newly diagnosed patients and those resistant and/or intolerant to prior TKI therapy.^6^, ^9^, ^10^, ^11^ Current practice guidelines in Japan recommend imatinib, nilotinib, and dasatinib for newly diagnosed patients with CML in chronic phase (CML‐CP).^6^ For patients resistant or intolerant to first‐line TKIs, Japanese practice guidelines recommend switching to a different TKI with a non‐overlapping resistance profile to the first‐line agent, including any second‐generation TKI not already used, bosutinib, or ponatinib.^6^ Previous studies conducted in Japanese patients with CML‐CP who are treatment‐naive or resistant and/or intolerant to a first‐line TKI demonstrated reliable outcomes with second‐generation TKIs and the third‐generation TKI ponatinib.^9^, ^11^, ^12^, ^13^, ^14^, ^15^ However, with each additional line of treatment using currently available TKIs, the incidence of treatment failure and disease progression increases.^10^, ^16^, ^17^ Thus, there is a need for effective therapy options for patients with CML who have failed previous treatment with currently approved TKIs.^3^, ^4^

Asciminib, a novel, first‐in‐class BCR‐ABL1 inhibitor that acts by STAMP (specifically targeting the ABL myristoyl pocket), has a mechanism of action distinct from approved ATP‐competitive TKIs.^18^, ^19^, ^20^ Asciminib allosterically inhibits BCR‐ABL1 by binding to the myristoyl pocket of ABL1, thereby stabilizing an inactive conformation of the ABL kinase.^18^, ^19^, ^20^ This unique mode of action allows asciminib to maintain activity against most ABL1 kinase mutations that confer resistance to approved ATP‐competitive TKIs.^18^, ^21^

In phase 1, first‐in‐human, dose‐escalation study in heavily pre‐treated patients with CML‐CP, asciminib (10–200 mg once daily [qd] or twice daily [bid]) showed clinically meaningful efficacy, including a major molecular response rate (MMR; *BCR::ABL1* transcript levels on the international scale [*BCR::ABL1* ^IS^] ≤0.1%) by 12 months of 48% (44 of 91 evaluable patients), and a favorable safety and tolerability profile.^22^ This study also demonstrated clinical efficacy and a favorable safety profile of asciminib at 200 mg bid in patients with CML‐CP/AP harboring the T315I mutation, with 23 of 49 patients (46.9%) achieving MMR by week 96.^22^, ^23^

Asciminib was further investigated in the ASCEMBL study (NCT03106779), an ongoing, international, open‐label, randomized, active‐controlled, phase 3 study in patients with CML‐CP (not harboring the *BCR::ABL1* T315I or V299L mutations) who were previously treated with ≥2 TKIs.^24^ In the primary analysis of the ASCEMBL study, asciminib (40 mg bid; *n* = 157) demonstrated statistically significant and clinically meaningful superiority in efficacy vs the second‐generation ATP‐competitive TKI bosutinib (500 mg qd; *n* = 76), with a larger proportion of patients achieving the primary endpoint of MMR at week 24 (25.5% vs. 13.2%; difference: 12.2% after adjusting for major cytogenetic response [MCyR; 0 to 35% Ph + metaphases] status at baseline; 95% confidence interval (CI), 2.19–22.3; *p* = 0.029).^24^ The safety profile of asciminib was favorable relative to that of bosutinib, as indicated by lower incidences of grade ≥ 3 adverse events (AEs) (50.6% vs. 60.5%) and AEs leading to treatment discontinuation (5.8% vs. 21.1%).^24^ After an additional 5 months of follow‐up in ASCEMBL, asciminib continued to demonstrate a sustained superior efficacy compared with bosutinib, with a consistently higher MMR rate (cumulative incidence of MMR by week 48, 33.2% vs. 18.6%) and a limited adverse event profile.^25^

Recently, asciminib has been approved by the US Food and Drug Administration (FDA; October 2020) for treatment of patients with Ph‐positive (Ph+) CML‐CP, previously treated with ≥2 TKIs and for adults with Ph + CML‐CP harboring the T315I mutation^26^ and by the Ministry of Health, Labour, and Welfare in Japan for treatment of patients with resistance or intolerance to previous TKI therapy.^27^

Japan is one of the several countries participating in the ASCEMBL study. In this report, we present the week 24 results of a subgroup analysis of Japanese patients enrolled in the ASCEMBL study, which aimed to determine if results in this subpopulation are comparable to those from the global study population. As only a small number of Japanese patients were enrolled in the bosutinib arm, this report focuses mainly on the efficacy and safety of asciminib in this patient subgroup.

## 2 METHODS

### 2.1 Ethics

The ASCEMBL study was conducted in accordance with the principles of the Declaration of Helsinki. An independent ethics committee or institutional review board at each center approved the study protocol. All patients provided written informed consent before the study initiation.

### 2.2 Study design and patients

**FIGURE 1:**
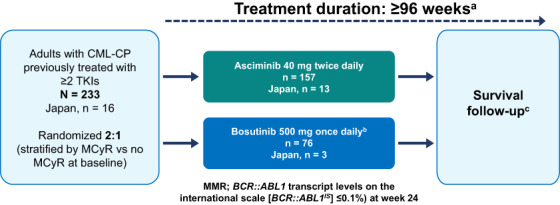
ASCEMBL study design. CML‐CP, chronic myeloid leukemia in chronic phase; FPFV, First patient first visit; MCyR, major cytogenetic response; TKI, tyrosine kinase inhibitors. ^a^Patients will continue to receive study treatment for up to 96 weeks after the last patient's first dose or 48 weeks after the last patient switches to asciminib, whichever is longer; ^b^Option of switch to asciminib allowed only for patients meeting lack of efficacy criteria based on ELN 2013 recommendations; ^c^Survival follow‐up is not part of this report.

ASCEMBL is an ongoing, phase 3, global, multi‐center, open‐label, randomized, active‐controlled study (Figure 1). Patients were randomized (2:1), to receive either asciminib 40 mg bid or bosutinib 500 mg qd. The randomization was carried out using a computer‐generated list and stratified by the presence or absence of MCyR at baseline. This manuscript presents the results of patients randomized in 10 sites in Japan in the ASCEMBL study. Patients were recruited from October 2017 (April 2018 for Japanese sites) until December 2019. Data cut‐off date for the primary analysis, including this subgroup analysis, was May 25, 2020 (when all patients had completed the week 24 visit or discontinued before). Full details of the ASCEMBL study design, patient inclusion, and exclusion criteria, and study assessments have been published previously.^24^ In brief, the study included patients aged ≥18 years with CML‐CP who had been previously treated with ≥2 TKIs and who had experienced at screening treatment failure or intolerance to the most recent TKI therapy. Treatment failure (lack of efficacy) was defined per European LeukemiaNet (ELN) 2013 recommendations for patients receiving a second line TKI^3^; intolerance was defined as nonhematologic grade 3 or 4 AEs while on treatment; persistent grade 2 AEs which are unresponsive to optimal management including dose adjustments; or hematologic grade 3 or 4 AEs while on treatment which are recurrent after dose reduction to the lowest recommended dose. At screening, *BCR::ABL1* ^IS^ was required to be ≥1%, except in patients with intolerance to their most recent TKI who were required to have *BCR::ABL1* ^IS^ of >0.1% at screening.

Key exclusion criteria were the presence of T315I or V299L mutations (i.e., known bosutinib‐resistant *BCR::ABL1* mutations) at any time prior to study entry; known second CML‐CP after previous progression to acute phase/blast crisis; treatment history with hematopoietic stem‐cell transplantation (HSCT) or planning to undergo allogeneic HSTC; or presence or history of cardiac or cardiac repolarization abnormality. Patients meeting lack of efficacy criteria as per ELN 2013 recommendations after randomization in either treatment arm were required to permanently discontinue study treatment.

Patients who failed bosutinib based on efficacy (based on 2013 ELN guidelines) could switch to asciminib within 96 weeks after the last patient completed the randomized study treatment (protocol amendment; December 14, 2018). Bosutinib patients were not allowed to switch to asciminib if they discontinued bosutinib due to intolerance or for any reasons other than lack of efficacy. Data collected for patients receiving asciminib after switching from bosutinib are not included in the present analysis.

### 2.3 Treatments

Asciminib and bosutinib were taken orally as tablets. Asciminib was taken in the fasted state (fasting for at least 2 h before and at least 1 h after taking asciminib; water was permitted); bosutinib was taken with food. Concomitant medications deemed necessary for the supportive care of patients were permitted. Treatment was continued for up to 96 weeks after the last patient received the first dose of study treatment or up to 48 weeks after the last bosutinib patient has switched to asciminib (whichever occurs last).

### 2.4 Endpoints

The primary endpoint in the ASCEMBL study was the rate of MMR (*BCR::ABL1* ^IS^ ≤0.1%) at week 24^25^ without having met treatment failure criteria before week 24. Secondary endpoints reported in this study included time to MMR, duration of MMR, *BCR::ABL1* ^IS^ ≤1% at week 24, molecular response rate 4 (MR^4^; *BCR::ABL1* ^IS^ ≤0.01%) at week 24, molecular response rate 4.5 (MR^4.5^; *BCR::ABL1* ^IS^ ≤0.0032%) at week 24, and complete cytogenetic response (CCyR; 0% Ph + metaphases) at week 24.^24^

### 2.5 Study assessments

Assessments of molecular, cytogenetic, and hematologic responses were performed as described previously^24^ and are briefed in Supplement section.

### 2.6 Statistical analysis

All Japanese patients who were randomized in the ASCEMBL study were included in the efficacy analysis (full analysis set, FAS). All Japanese patients who received at least one dose of study drug were included in the safety analysis (safety set). For efficacy endpoints, the rate and associated 95% CI (based on the Clopper‐Pearson method) were presented for each treatment group. Owing to the limited number of patients in this subgroup analysis, no statistical testing was performed between treatment arms, and results are presented by descriptive statistics only. AEs were coded using the MedDRA terminology (Version 23.0) and assessed as per the Common Terminology Criteria for Adverse Events (CTCAE) version 4.03.

## 3 RESULTS

### 3.1 Participants' disposition

Overall, 16 patients in 10 sites in Japan were randomized in the ASCEMBL study to receive asciminib (*n* = 13) or bosutinib (*n* = 3) (Table 1). At the time of data cut‐off, randomized treatment was ongoing for 11 patients (84.6%) in the asciminib group and for none of the patients in the bosutinib group. Two patients (15.4%) in the asciminib group discontinued asciminib treatment after week 24 and prior to week 48 due to lack of efficacy and adverse event, respectively. In the bosutinib group, 2 of the 3 patients discontinued bosutinib before week 24 due to adverse events (diffuse large B‐cell lymphoma and neutropenia, and drug eruption); the third patient met failure criteria (lack of efficacy) at week 24 and switched to asciminib.

**TABLE 1:** Patient disposition

Patients, *n* (%)	Japanese subgroup	
	Asciminib	Bosutinib
	(*n* = 13)	(*n* = 3)
Treated	13 (100)	3 (100)
Treatment ongoing^a^	11 (84.6)	0
Discontinued from treatment	2 (15.4)	3 (100)
<Week 24	0	2 (66.7)
≥Week 24 and <Week 48	2 (15.4)	1 (33.3)
Reason for discontinuation		
Lack of efficacy	1 (7.7)	1 (33.3)
Adverse event	1 (7.7)	2 (66.7)
Switched to receive asciminib^b^	NA	1 (33.3)

Abbreviation: NA, not applicable.

^a^ Ongoing at the time of data cutoff: May 25, 2020.

^b^ Patients could switch to asciminib upon meeting lack of efficacy criteria as per ELN 2013 recommendations on bosutinib.

### 3.2 Baseline characteristics ‐ asciminib group

Baseline characteristics of patients randomized to asciminib are shown in Table 2. The baseline median age was 56 years (range, 28–70), and most patients were male (69.2%). The most common reasons for discontinuation of the last prior TKI were lack of efficacy (*n* = 7 [53.8%]) and lack of tolerability (*n* = 6 [46.2%]). At baseline, 9 (69.2%) asciminib‐randomized patients were in MCyR, and the majority of patients (7 [53.8%]) had *BCR::ABL1* ^IS^ >1 to ≤10%. No patient had any *BCR::ABL1* mutation.

**TABLE 2:** Demographics and baseline characteristics

Variable	Japanese subgroup	
	Asciminib	Bosutinib
	(*n* = 13)	(*n* = 3)
Median age, years (range)	56.0 (28–70)	40.0 (27–66)
Female, *n* (%)	4 (30.8)	3 (100)
Male, *n* (%)	9 (69.2)	0
MCyR, *n* (%)	9 (69.2)	1 (33.3)
Reason for discontinuation of last TKI, *n* (%)		
Lack of efficacy	7 (53.8)	2 (66.7)
Lack of tolerability	6 (46.2)	1 (33.3)
Number of lines of prior TKI therapy, *n* (%)		
2	5 (38.5)	1 (33.3)
3	6 (46.2)	1 (33.3)
4	2 (15.4)	0
≥5	0	1 (33.3)
*BCR::ABL1* ^IS^ at baseline, *n* (%)		
>0.1% to ≤1%	3 (23.1)	0
>1% to ≤10%	7 (53.8)	0
>10%	3 (23.1)	3 (100)
Patients with any *BCR::ABL1* mutation, *n* (%)	0	0

Abbreviations: IS, international scale; MCyR, major cytogenetic response; TKI, tyrosine kinase inhibitor.

### 3.3 Efficacy ‐ asciminib group

**FIGURE 2:**
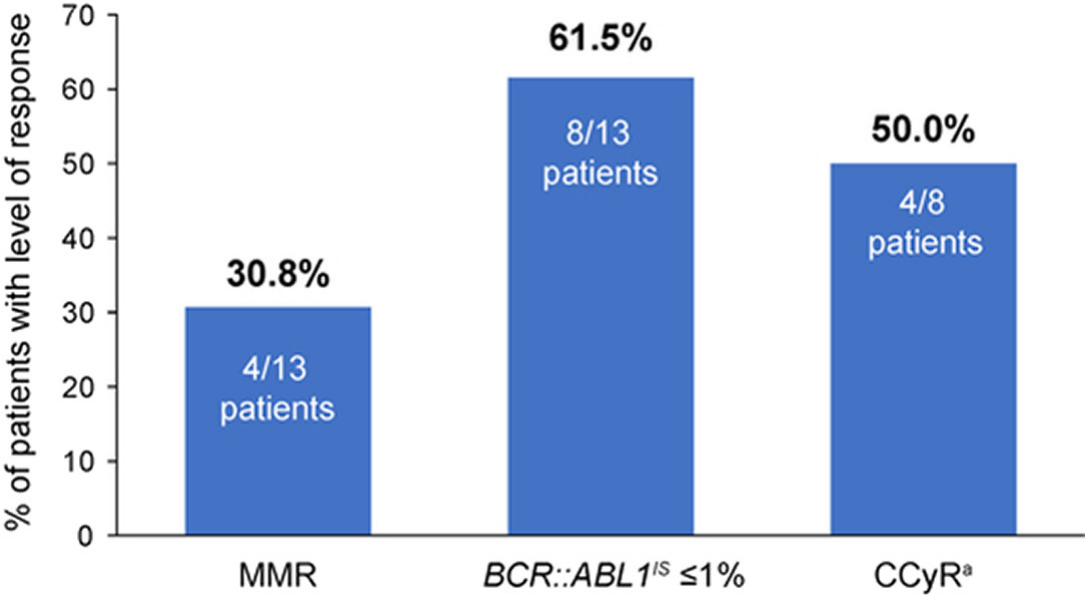
Efficacy endpoints at week 24 in Japanese patients randomized to Asciminib in the ASCEMBL study. ^a^Among patients without CCyR at baseline (*n* = 8); CCyR, complete cytogenetic response; IS, international scale; MMR, major molecular response (*BCR::ABL1* ^IS^ ≤0.1%). In the bosutinib group (*n* = 3), no patient achieved MMR, *BCR::ABL1* ^IS^ ≤1% or CCyR at week 24.

Efficacy results of patients randomized to asciminib are shown in Figure 2 and Table S1. At week 24, the MMR rate (primary endpoint in the primary analysis of the ASCEMBL study) with asciminib was 30.8% (4/13; 95% CI, 9.09–61.43). The cumulative MMR by week 24 was 30.8% (95% CI, 9.09–61.43). Among patients who achieved MMR, the median time to first MMR was 12.1 (range 4–36) weeks. No patient had lost MMR by the time of data cut‐off. One patient (1/13; 7.7%) achieved a deeper molecular response of MR^4.5^ (*BCR::ABL1* ^IS^ ≤0.0032%) at week 24. At week 24, 61.5% (8/13) of patients had *BCR::ABL1* ^IS^ ≤1% regardless of baseline *BCR::ABL1* ^IS^ levels. Among patients without CCyR at baseline, the CCyR rate at week 24 was 50.0% (4/8; 95% CI, 15.70–84.30).

### 3.4 Baseline characteristics and efficacy ‐ bosutinib group

Description of the baseline characteristics (Table 2) and efficacy outcomes (Table S1) in the bosutinib group are limited by the low number of Japanese patients randomized to bosutinib (*n* = 3). At baseline, all 3 patients had *BCR::ABL1* ^IS^ > 10%, and for none of the three patients any *BCR::ABL1* mutations were identified. At week 24, no patient was in MMR and no patient had *BCR::ABL1* transcript level ≤1%. None of the two patients without CCyR at baseline had CCyR at week 24. One patient switched to asciminib at week 24 after meeting lack of efficacy criteria with *BCR::ABL1* ^IS^ > 10% at week 24.

### 3.5 Safety

Median duration of exposure to study treatment was 44.9 (range, 20.1–101.6) weeks for asciminib and 3.1 (range, 1.3–29.0) weeks for bosutinib. Overall, 10 (76.9%) patients in the asciminib group, and 3 (100.0%) patients in the bosutinib group experienced at least 1 AE (any grade) during the study (Table S2). Neutropenia was the most common AE observed with asciminib (38.5%), whereas diarrhea was the most common AE observed with bosutinib (100.0%) (Table 3). No SAEs were reported in the asciminib group, whereas two (66.7%) out of the three patients in the bosutinib group reported SAEs. Grade ≥ 3 AEs were reported in four (30.8%) and two (66.7%) patients belonging to asciminib and bosutinib groups, respectively (Table S2). Treatment‐related AEs were reported by six (46.2%) and three (100.0%) patients in the asciminib and bosutinib groups, respectively. AEs leading to treatment discontinuation were observed in one (7.7%) and two patients (66.7%) in the asciminib and bosutinib groups, respectively. Five (38.5%) patients in the asciminib arm and two (66.7%) patients in the bosutinib arm had ≥1 dose adjustment and/or interruption due to AEs. No deaths were reported during the study (Table S2).

**TABLE 3:** Adverse events occurring in ≥2 patients

Event, *n* (%)	Japanese subgroup			
	Asciminib		Bosutinib	
	(*n* = 13)		(*n* = 3)	
	All grades	Grade ≥ 3	All grades	Grade ≥ 3
Number of patients with ≥1 adverse event	10 (76.9)	4 (30.8)	3 (100)	2 (66.7)
Nasopharyngitis	4 (30.8)	0	0	0
Neutrophil count decreased	3 (23.1)	3 (23.1)	1 (33.3)	0
Diarrhea	1 (7.7)	0	3 (100)	0
Platelet count decreased	3 (23.1)	2 (15.4)	0	0
Neutropenia	2 (15.4)	1 (7.7)	1 (33.3)	1 (33.3)
Constipation	0	0	2 (66.7)	0

*Note*: Numbers represent counts of patients. A patient with multiple severity grades for an adverse event is only counted under the maximum grade; MedDRA version 23.0, CTCAE version 4.03.

Grade ≥ 3 clinical laboratory or electrocardiogram abnormalities observed with asciminib included decreased neutrophils (23.1%), decreased platelets (1.7%), decreased leukocytes (1.7%), and ECG QT prolongation (1.7%), and only increased ALT (33.3%) was observed with bosutinib.

## 4 DISCUSSION

ASCEMBL is the first randomized, controlled study in patients with CML‐CP who have been previously treated with TKIs. In the primary analysis of the overall study population, asciminib demonstrated a statistically significant clinical benefit versus bosutinib, meeting the primary study endpoint with an almost two times higher 24‐week MMR rate in asciminib‐randomized patients vs bosutinib‐randomized patients.^24^

The present study is the first report providing evidence on the efficacy and safety of asciminib in patients from Japan. In Japanese patients, MMR rates achieved with asciminib at week 24 (30.8%) were comparable to those observed with asciminib in the overall ASCEMBL study population (25.5%; Table 4).^24^ Likewise, efficacy results were similar or slightly higher in the Japanese subgroup compared with the overall ASCEMBL study population for median time to achieve first MMR (12.1 vs. 12.7 weeks) and for the week 24 endpoints CCyR (50.0% vs. 40.8%), MR^4^ and MR^4.5^ (7.7% vs. 10.8% and 7.7% vs. 8.9%, respectively), and the proportion of patients with *BCR::ABL1* ^IS^ ≤ 1% (61.5% vs 49.0%; Table 4).^24^

**TABLE 4:** Week 24 efficacy and safety in Japanese patients and in all patients randomized to asciminib in the ASCEMBL study

*n* (%)	ASCEMBL	
	Japanese patients randomized to asciminib (*n* = 13)	All patients randomized to asciminib^24^ (*n* = 157)
Molecular response at week 24		
*BCR::ABL1* ^IS^ ≤ 1%	8 (61.5)	77 (49.0)
MMR (*BCR::ABL1* ^IS^ ≤0.1%)	4 (30.8)	40 (25.5)
MR^4^ (*BCR::ABL1* ^IS^ ≤0.01%)	1 (7.7)	17 (10.8)
MR^4.5^ (*BCR::ABL1* ^IS^ ≤0.0032%)	1 (7.7)	14 (8.9)
Cytogenetic response at week 24		
CCyR^a^	4 (50.0)	42 (40.8)
Safety		
AEs	10 (76.9)	140 (89.7)
Grade ≥ 3 AEs	4 (30.8)	79 (50.6)
Treatment‐related AEs	6 (46.2)	99 (63.5)
SAEs	0	21 (13.5)
Fatal SAEs	0	2 (1.3)
AEs leading to discontinuation	1 (7.7)	9 (5.8)
AEs leading to dose adjustment/interruption	5 (38.5)	59 (37.8)
AEs requiring additional therapy	9 (69.2)	103 (66.0)

Abbreviations: CCyR, complete cytogenetic response; IS, international scale; MMR, major molecular response; MR^4^, molecular response 4 (*BCR::ABL1* ^IS^ ≤ 0.01%); MR^4.5^, molecular response 4.5 (*BCR::ABL1* ^IS^ ≤ 0.0032%).

^a^ Among patients without CCyR at baseline (Japan *n* = 8; All patients *n* = 103).

In the present subgroup analysis, no patient receiving bosutinib achieved MMR, CCyR, or *BCR::ABL1* ^IS^ ≤ 1% at week 24. However, there were only three patients in the bosutinib group, which limits the generalizability of these results and does not allow a conclusion to be drawn from this subgroup.

In the overall ASCEMBL study population, the difference in MMR rates at week 24 between asciminib and bosutinib was 12.2%. Of note, a higher MMR rate was achieved with asciminib vs bosutinib, irrespective of prior lines of therapy, and in patients who discontinued their previous TKI treatment due to efficacy reasons.^24^ The finding that asciminib is associated with the achievement of MMR in a considerable proportion of patients failing prior lines of TKI therapy is clinically relevant because after resistance to a second‐generation TKI, a different second‐generation TKI can be used but generally does not lead to deep and durable responses.^28^

No comparative conclusions for asciminib versus bosutinib can be drawn from this Japanese subgroup analysis due to the small number of bosutinib patients.

The safety profile of asciminib observed in this Japanese subgroup is comparable to that observed in the overall ASCEMBL study population and is also in line with that observed in the phase 1 study.^22^, ^24^ By the time of data cut‐off, no deaths were reported in the Japanese subgroup, and the safety data did not reveal any specific safety finding for Japanese patients on asciminib in this study. Neutropenia and thrombocytopenia were the most common AEs observed with asciminib in this Japanese subgroup as well as in the overall study population. Of note, nausea, which was reported in 11.5% of patients in the overall ASCEMBL study population, was not reported in this Japanese subgroup.

With ATP‐competitive inhibitors such as imatinib and second‐generation TKIs, many AEs are due to off‐target activities as these agents are not specific for ABL1, ABL2, and BCR‐ABL1.^29^, ^30^ Conversely, by specifically targeting the myristoyl pocket of BCR‐ABL1, asciminib has the potential for a more favorable safety profile than ATP‐competitive inhibitors, whilst demonstrating robust efficacy for the treatment of patients with CML‐CP who are resistant or intolerant to currently approved TKIs.

In ASCEMBL, the starting dose of bosutinib was 500 mg qd, which is the approved dose for Japanese patients with resistant and/or intolerant CML.^31^, ^32^ In current practice, to improve the tolerability of bosutinib some patients are resistant or intolerant to previous treatments may use lower starting doses (e.g., 400 mg qd). However, a recent pooled analysis of bosutinib studies comparing Japanese versus non‐Japanese patients have not shown improved tolerability of bosutinib used at a starting dose of 400 mg qd as compared to 500 mg qd.^33^ In Japanese patients with newly diagnosed CML (starting dose 400 mg qd) the permanent discontinuation rate was 32% while in Japanese patients treated in second or later lines of therapy (starting dose 500 mg qd) it was 28.6%.^33^ In ASCEMBL, the dose modifications for toxicities were similar between treatment arms and comparable to the prescribing information for bosutinib. Further studies may be required to assess bosutinib outcomes using alternative dosing schemes in patients with R/I CML.

## 5 CONCLUSION

Efficacy and safety results observed with asciminib in this Japanese subgroup are comparable to those previously reported for the overall study population in the ASCEMBL study. The findings support asciminib as a new therapeutic option for Japanese patients with CML‐CP after prior treatment with TKIs.

## AUTHOR CONTRIBUTIONS

All authors were involved in the designing of the study, collection, analysis, and interpretation of data. All authors contributed to the drafting and reviewing of the manuscript and provided final approval of the version to be published.

## FUNDING INFORMATION

This study was sponsored by Novartis Pharma K.K.(Japan).

## CONFLICT OF INTEREST

Naoto Takahashi has received honoraria from Pfizer, Otsuka, and Novartis; research funding from Pfizer, Otsuka, Novartis, Chugai, Eizai, Asahikasei, Ono, and Kyowahakko‐Kirin outside the submitted work. Yosuke Minami received research funding from Ono and CMIC, and honoraria from Bristol‐Myers Squibb, Novartis, Astellas, and Daiichi‐Sankyo. Itaru Matsumura received honoraria from Novartis Pharma KK, Bristol‐Myers Squibb, Pfizer Japan Inc., Daiichi Sankyo Co Ltd., Otsuka Pharmaceutical Co Ltd., Astellas Pharma Inc., Amgen Astellas BioPharma K.K., Janssen Pharmaceutical K.K., AbbVie GK.; research funding from Chugai Pharmaceutical Co., Ltd., Novartis Pharma KK, AbbVie GK., Takeda Pharmaceutical Company Limited., Pfizer Japan Inc., Eisai Co., Ltd., Alexion, Asahikasei, Ono, Kyowahakko‐Kirin, Shionogi & Co., Ltd., Sumitomo Dainippon Pharma Co., Ltd., Nippon Shinyaku Co., Ltd., Taiho Pharmaceutical Co., Ltd., Mitsubishi Tanabe Pharma Corporation, Sanofi K.K., Astellas Pharma Inc., Otsuka Pharmaceutical Co., Ltd, MSD K.K., outside the submitted work. Hiroshi Matsuoka received research funding from Takeda Pharmaceutical Company Limited, Sysmex Corporation. Takafumi Yokota is an employee of Teijin Limited and received research funding from LUCA Science Inc. Akihiro Tomita received research funding from Perseus Proteomics Inc, Novartis Pharma K.K., Pfizer Japan Inc, Chugai Pharmaceutical Co., Ltd., Kyowa Kirin Ltd., Ono Pharmaceutical Co., Ltd., and Taiho Pharmaceutical Co., Ltd. Akio Maki, Makoto Aoki, and Alex Allepuz are employees of Novartis. Noriko Doki, Kohmei Kubo, Keita Kirito, Tatsunori Goto, and Junichiro Yuda have nothing to disclose.

## Supporting information


Appendix S1

## Data Availability

Novartis is committed to sharing with qualified external researchers access to patient‐level data and supporting clinical documents from eligible studies. These requests are reviewed and approved by an independent review panel based on scientific merit. All data provided are anonymized to respect the privacy of patients who have participated in the trial consistent with applicable laws and regulations. This trial data availability is according to the criteria and process described on www.clinicalstudydatarequest.com.
