# Supplementary material for: Asciminib vs bosutinib in CML patients pretreated with ≥2 tyrosine kinase inhibitors: Results from the Japanese subgroup analysis of ASCEMBL study
Source: Cancer Med. 2022 Sep 27;12(3):2990–8. doi: 10.1002/cam4.5212 (PMC9939084; doi:10.1002/cam4.5212)
Supplement: Supplementary file 1 — Appendix S1 [file CAM4-12-2990-s001.pdf]

SUPPLEMENT

Table S1. Comparison of Molecular and Cytogenic Responses in Japanese Patients Randomized to Asciminib vs. Bosutinib at Week 24.

n (%)	Japanese subgroup	
	**Asciminib (n=13)**	**Bosutinib (n=3)**
**Molecular response at week 24**		
MMR (*BCR::ABL1*^{IS} ≤0.1%)	4 (30.8)	0 (0)
BCR::ABL1^{IS} ≤ 1%	8 (61.5)	0 (0)
MR^4 (*BCR::ABL1*^{IS} ≤ 0.01%)	1 (7.7)	0 (0)
$MR^{4.5}$ (*BCR::ABL1*^{IS} ≤ 0.0032%)	1 (7.7)	0 (0)
**Cytogenetic response at week 24**		
CCyR[a]	4 (50.0)	0 (0)

[a]Among patients without CCyR at baseline (asciminib n=8; bosutinib n=2); CCyR, complete cytogenetic response; IS, international scale; MMR, major molecular response; MR^4, molecular response 4 (*BCR::ABL1*^{IS} ≤ 0.01%); $MR^{4.5}$, molecular response 4.5 (*BCR::ABL1*^{IS} ≤ 0.0032%)

For Review Only

Table S2. Overview of Adverse Events

Category, n (%)*	Japanese Subgroup			
	**Asciminib (n=13)**		**Bosutinib (n=3)**	
	All grades	Grade ≥3	All grades	Grade ≥3
AEs	10 (76.9)	4 (30.8)	3 (100)	2 (66.7)
SAEs	0	0	2 (66.7)	1 (33.3)
Fatal SAEs	0	0	0	0
AEs leading to discontinuation	1 (7.7)	1 (7.7)	2 (66.7)	1 (33.3)
AEs leading to dose adjustment/interruption	5 (38.5)	4 (30.8)	2 (66.7)	2 (66.7)
AEs requiring additional therapy	9 (69.2)	2 (15.4)	3 (100)	2 (66.7)

Numbers represent counts of patients. A patient with multiple severity grades for an adverse event is only counted under the maximum grade; MedDRA version 23.0, CTCAE version 4.03.
AE, adverse event; SAE, serious AE

For Review Only

Study assessments

Assessments of molecular, cytogenetic and hematologic responses were performed as described previously.[26] Molecular response was assessed based on levels of *BCR::ABL1* transcripts that were determined by real-time quantitative PCR testing of peripheral blood and analyzed at a central testing laboratory. Blood samples (20 mL) for *BCR::ABL1* quantification were collected at screening and at weeks 4, 8, 12, 16, and 24 and every 12 weeks thereafter until EOT. Blood samples (5 mL) for *BCR::ABL1* mutation analysis by Sanger sequencing was collected on day 1 of week 1 and upon confirmed loss of MMR and/or at EOT. For patients with mutations on day 1 of week 1, mutation analysis was repeated at week 12 visit and every 12 weeks thereafter up to EOT.

Bone marrow aspirate for cytogenetic analyses were to be performed at screening (performed up to 56 days prior to Week 1 Day 1) and at Week 24, 48, 72, 96 as long as patient had not achieved MMR, and at EOT. Cytogenetic response was assessed locally as the percentage of Ph+ metaphases in the bone marrow with at least 20 metaphases examined.

For hematologic assessments, blood samples were collected at screening; at the week 1, day 1 visit; at the week 2, day 1 visit; every 2 weeks from week 4 up to the week 16 visit; every 4 weeks up to the week 96 visit and every 12 weeks thereafter; at the EOT; and as clinically indicated. Hematologic parameters were assessed by a central laboratory, except for samples collected at weeks 6, 10, and 14 which were assessed on-site or at a local laboratory.

For safety assessments, patients to have study visits off-site (e.g., by telephone or video consultation with site study staff) or home visit by site study staff (if locally applicable). Laboratory tests, ECGs, imaging were performed at a central laboratory or a local facility. All collected ECGs were reviewed using an independent central reader (Electronic Research Technology, Inc.).

For Review Only
